# The absolute and relative changes in high-sensitivity cardiac troponin I are associated with the in-hospital mortality of patients with fulminant myocarditis

**DOI:** 10.1186/s12872-021-02386-8

**Published:** 2021-11-30

**Authors:** Chao Liu, Zhongqin Wang, Kengquan Chen, Guanglin Cui, Chen Chen, Luyun Wang, Jiangang Jiang

**Affiliations:** Division of Cardiology, Department of Internal Medicine, and Hubei Key Laboratory of Genetics and Molecular Mechanisms of Cardiological Disorders, Tongji Hospital, Tongji Medical College, Huazhong University of Science and Technology, Wuhan, 430030 China

**Keywords:** Fulminant myocarditis, High-sensitivity troponin I, Absolute change, Relative change, In-hospital mortality

## Abstract

**Background:**

We sought to describe the tendency and extent of high-sensitivity cardiac troponin I (hs-cTnI) changes in patients with fulminant myocarditis (FM) after admission and to explore the relationship between the in-hospital mortality of FM and the absolute and relative changes in hs-cTnI within 24 h and 48 h after admission.

**Methods:**

In the retrospective study, the object are patients diagnosed with FM in our single centre. The value of cardiac troponin was recorded after patients admitted to hospital in succession. The absolute and relative changes in hs-cTnI within 24 h and 48 h were described as range distributions. Receiver operating characteristic (ROC) curve and Cox analyses were performed to determine the relationship between in-hospital mortality of FM and hs-cTnI changes.

**Results:**

A total of 83 FM patients admitted to our centre from January 1, 2010 to December 31, 2019 were included; 69 patients survived and 14 patients died. In the survival group, 78% of patients experienced a decline in hs-cTnI within 24 h, while 36% of the mortality group exhibited a declining tendency in hs-cTnI (*P* = 0.003). Nearly 60% of survival group had a 0–2000 ng/l reduction in troponin from baseline within 24 h of admission. However, troponin levels of 50% of patients in the mortality group were 0–10,000 ng/ L higher than baseline 24 h after admission. Multivariable logistic analysis revealed that the declining tendency of hs-cTnI within 24 h, in addition to time from onset to admittance to hospital, intravenous immunoglobulin treatment and the abnormal level of creatinine, were associated with the in-hospital mortality of FM (for the declining tendency of hs-cTnI within 24 h, OR = 0.10, 95% CI 0.02–0.68, *P* = 0.018). The ROC curve revealed optimal cut-off values of − 618 ng/l for absolute change within 24 h (AUC = 0.800, *P* < 0.01), − 4389 ng/l for absolute change within 48 h (area under the curve = 0.711, *P* < 0.01), − 28.46% for relative change within 24 h (AUC = 0.810, *P* < 0.01), and − 52.23% for relative change within 48 h (AUC = 0.795, *P* < 0.01). Absolute changes and relative changes in hs-cTnI within 24 h and 48 h were strong predictors of in-hospital mortality by Cox regression analysis after adjustment for sex, time from onset to admission, and occurrence of ventricular tachycardia or ventricular fibrillation.

**Conclusion:**

Most FM patients who survived experienced a decline in hs-cTnI within 24 h. The absolute and relative changes in hs-cTnI within 24 h and 48 h were strong predictors of in-hospital mortality.

**Supplementary Information:**

The online version contains supplementary material available at 10.1186/s12872-021-02386-8.

## Introduction

Myocarditis is an inflammatory disease of the myocardium that has a broad spectrum of clinical presentations, ranging from mild symptoms to life-threatening arrhythmias and/or severe heart failure (HF) [1]. Fulminant myocarditis (FM) is a rare but most severe type of myocarditis and is characterized by sudden occurrence, rapid progression, and haemodynamic dysfunction [2]. Although endomyocardial biopsy (EMB) is the gold standard for myocarditis diagnosis, it is pragmatic to determine the treatment course for patients suspected of having myocarditis through the use of clinical diagnosis based on the clinical state as well as laboratory and imaging tests [2, 3]. Specifically, FM can be clinically defined as a distinct onset of symptoms in the prior 2 weeks, severe symptoms of HF, and hypotension or overt cardiogenic shock requiring inotropes, vasopressors, and/or mechanical circulation support [3]. As the technology advanced, gadolinium contrast-enhanced cardiac magnetic resonance (CMR) can afford tissue-level pathologies consistent with myocarditis, including myocardial oedema and fibrosis (e.g., T2- and T1-weighted sequences and late gadolinium enhancement [LGE]), which has high diagnostic accuracy [4, 5].

Myocarditis is an inflammatory cardiac disorder induced predominantly by viruses [6]. The mechanisms of myocardial injury are recognized as direct injury and indirect immunogenic injury [7]. The former indicates intracellular viral replication in the myocardium and other tissues, resulting in degeneration, necrosis, and dysfunction [8]. The latter is triggered by cytotoxic and antigen–antibody reactions, which are caused by viral infection [9]. An elevated serum cardiac troponin (cTn) is almost always present in patients with FM [10]. Especially, the case for the assay of high-sensitivity troponin I (hs-cTnI) detection which is more sensitive in reflecting the potential injury of the myocardium [11]. Whereas normal levels of cardiac troponin do not exclude FM [10]. Research on the diagnostic and prognostic value of serial changes in hs-cTnI and elevated hs-cTnI has been widely performed in patients with myocardial infarction (MI) [12–17]. FM patients’ hs-cTnI kinetics in the early phase after admission and the relationship between serial changes in hs-cTnI and patient in-hospital mortality remain to be determined.

Therefore, the main purpose of this retrospective research is to describe the distribution of cardiac troponin concentrations and the change of cardiac troponin over time in FM patients, and to explore whether the troponin distribution and change over time differs between those who died during the initial hospital admission as compared to those who did not. Besides, we also explored the prognostic value of the absolute (Δ) and relative (Δ%) changes in hs-cTnI within 24 h and 48 h after admission for FM patient in-hospital mortality.

## Methods

### Study population and diagnosis of fulminant myocarditis

This is a retrospective, single centre study. All patients were in-patients at TongJi Hospital, a tertiary teaching medical centre in Wuhan, China from January 1, 2010 to December 31, 2019. We declare that the study is compliant with the rules and regulations outlined in the Declaration of Helsinki. Informed consent was obtained from all participants discharged. The study has been approved by the Tongji Hospital, Tongji Medical College, Huazhong University of Science and Technology Institutional Review Board (TJ-C20160202).

FM is usually defined as myocardial inflammatory disease with a rapid onset complicated with severe haemodynamic dysfunction. FM is more likely to be a clinical diagnosis rather than a histological or pathological diagnosis. Therefore, FM was diagnosed in our centre, predominantly as recommended by the Chinese expert consensus statement [2] according to the following signs: sudden attack, obvious premonitory symptoms of viral infection (especially severe fatigue and poor appetite), and rapidly emerging severe haemodynamic dysfunction, serious myocardial injuries, and diffuse ventricular wall motion decrease. Since 2018, CMR has been widely used in our centre for the diagnosis of FM, and 25 of 36 FM patients in that period underwent CMR examination. EMB was performed in 3 patients with unexplained heart failure as recommended by the guidelines [18]. Coronary angiography was performed in 36 cases to distinguish it from myocardial infarction according to clinical signs such as chest pain and elevated ST waves on electrocardiogram and elevated hs-cTnI [19]. All patients’ ultimate diagnoses were ascertained by at least two cardiologists before inclusion in the study.

### Data collection and hs-cTnI detection

All patients at admission underwent clinical assessments, including clinical history, physical examination, 12-lead electrocardiogram, echocardiography, and standard blood tests. Patients’ hospital outcomes were recorded. Clinical manifestations include time from onset to admission, complaint and premonitory symptoms, and ECG records of patients with arrhythmia and so on; Blood tests include blood routing tests, liver function and kidney function. Special examinations such as coronary angiography and cardiac magnetic resonance imaging, in addition with treatment patients received, were also recorded.

All hs-cTnI values measured within 7 days after admission were recorded. Considering the fact that the available hs-cTnI data we collected was concentrated in 24 h and 48 h after admission, thus, we choose to illustrate the absolute and relative changes in hs-cTnI in the period of 24 h and 48 h after admission. The level of hs-cTnI is tested by ARCHITECT i2000SR (Abbott Laboratories, Chicago, U.S.). This assay has a detection range of 1.9–50,000 ng/ L. It has an coefficient of variation of < 10% at 4.7 ng/L, with a limit of detection (LoD) of 1.9 ng/L and a limit of quantification (LoQ) of 3.5 ng/l. The assay has a 99th percentile URL of 28 ng/L, with sex-specific thresholds of 35 and 17 ng/L in men and women, respectively. According to the troponin detected after admission, the absolute value change (Δ) and relative ratio change (Δ%) of troponin for each patient were calculated respectively within 24 and 48 h after admission.

### Statistical analysis

The data are presented as proportions, mean ± SD, or medians with interquartile ranges if variables were nonnormally distributed. Comparisons were made with t-tests for normally distributed continuous variables, Mann–Whitney U tests for nonnormally distributed continuous variables, and χ^2^ tests for categorical variables. Kolmogorov–Smirnov test was used to assess the normality of the distribution. Receiver operating characteristic (ROC) curves were constructed to assess the relationship between the in-hospital outcome of FM patients and the absolute (Δ) and relative (Δ%) changes in hs-cTnI within 24 h and 48 h after admission. Optimal cut-off values were derived from ROC curves, and sensitivity and specificity were calculated. Kaplan–Meier curve was used to compare the in-hospital mortality in different groups divided by the cut-off point of the absolute (Δ) and relative (Δ%) changes in hs-cTnI within 24 h and 48 h.Cox regression analysis was performed to determine the association between the in-hospital mortality and kinetic change of hs-cTnI. All hypothesis testing was 2 tailed, and a *P* value of 0.05 was considered statistically significant. All statistical analyses were performed with SPSS 22.0 (SPSS Inc., Chicago, IL) for Windows (Microsoft Corp, Redmond, WA).

## Results

### Characteristics of FM patients

**Fig. 1:**
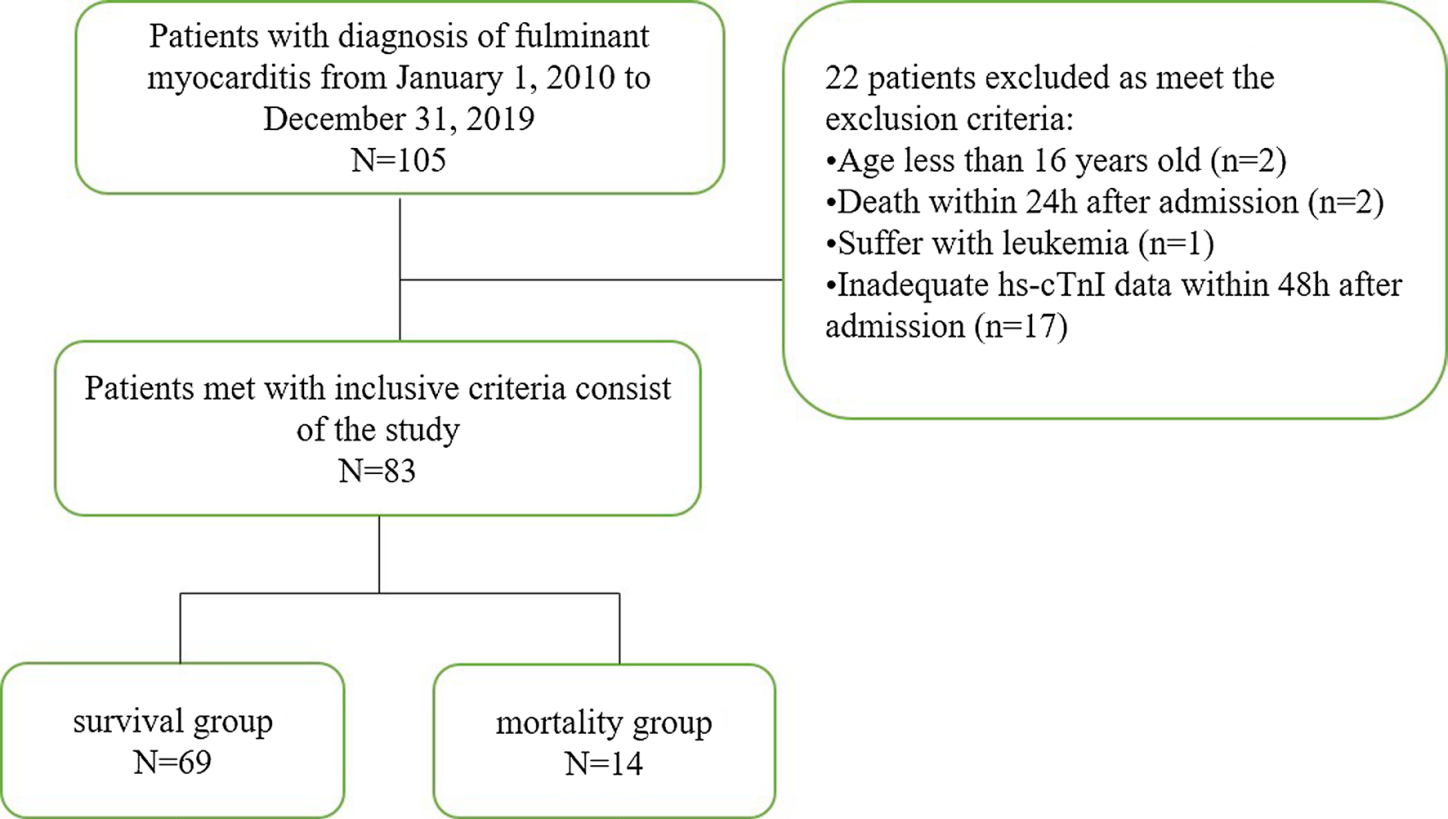
The study process describing enrolment of the patient cohort

A total of 105 FM cases were retrospectively included in the study, among which 22 cases were excluded (Fig. 1). The exclusion criteria were as follows: (1) age less than 16 years old, (2) diagnosis of other severe diseases, such as malignant tumours, (3) death within 24 h after admission to the hospital, and 4) lack of serial hs-cTnI records within 48 h. The baseline characteristics of all 83 FM patients are shown in Table 1. The median age was 37 (Q1–Q3: 29–48) years old, and 49.4% were women. Premonitoring symptoms of viral infection, such as fever, were observed in 75.9% of the total population, while coexisting conditions, including hypertension or diabetes mellitus, were observed in less than 10% of patients. The median time from onset to admission was 3 days, which indicated that FM was an acute disease; this period in the survival group was significantly shorter than that in the mortality group (*P* = 0.028). Chest distress (69.9%) was the most common symptom in FM patients, and chest pain (22.9%), palpitation (22.9%), dizziness (25.3%), and disturbances of consciousness (21.7%) were also recorded. The incidence of severe arrhythmia, such as ventricular tachycardia or ventricular fibrillation, was significantly higher in the mortality group than in the survival group (35.7% vs. 10.1%, *P* = 0.026). A higher level of creatinine but not alanine transaminase was also observed at admission in the mortality group than in the survival group (*P* = 0.016). Forty-two percent of the surviving group underwent CMR examination, and all of the examinations were performed in the last two years; no patients in the mortality group received such examinations as severe haemodynamic dysfunction. Coronary angiography was performed in 28.6% of the mortality group and 46.4% of the surviving group. Patients in the mortality group with rapid illness progression received vasoactive agents (100%), 50% of whom received intraaortic balloon pumps (IABPs) and mechanical ventilation therapy. The majority of patients (87%) in the survival group underwent IABP to maintain haemodynamic stability. Vasoactive agents were used in 66.7% of surviving patients, and mechanical ventilation was used in 36.2% of surviving patients. Approximately 20% of patients in both groups underwent extracorporeal membrane oxygenation (ECMO).

**Table 1:** Baseline characteristics of FM

	Total (n = 83)	Mortality (n = 14)	Survival (n = 69)	*P*
Female n;(%)	41 (49.4)	6 (42.9)	35 (50.7)	0.591
age (year) Median [Q1, Q3]	37 [29,48]	42.5 [32.75,50.75]	37 [27,47.5]	0.226
Premonitory symptoms of viral infection n;(%)	63 (75.9)	10 (71.4)	53 (76.8)	0.735
Co-existing conditions n;(%)				
Hypertension	6 (7.2)	1 (7.1)	5 (7.2)	1.000
Diabetes mellitus	4 (4.8)	0	4 (5.8)	1.000
Renal disease	1 (1.2)	1 (7.1)	0	0.169
Time from onset to admission (days) Median [Q1, Q3]	3 [2, 5]	4.5 [2.75,10.25]	3 [1, 4.5]	0.028
Coronary angiography (n, %)	36 (43.3)	4 (28.6)	32 (46.4)	0.220
CMR (n,%)	29 (34.9)	0	29 (42.0)	0.002
Clinical symptoms n;(%)				
Chest pain	19 (22.9)	2 (14.3)	17 (24.6)	0.506
Chest distress	58 (69.9)	10 (71.4)	48 (69.6)	1.000
Palpitation	19 (22.9)	3 (21.4)	16 (23.2)	1.000
Dizziness	21 (25.3)	2 (14.3)	19 (27.5)	0.501
Disturbances of consciousness	18 (21.7)	3 (21.4)	15 (21.7)	1.000
Ejection fraction at lowest (x ± s)	33.7 ± 13.5	40.9 ± 15.3	32.5 ± 13.0	0.056
ECG n;(%)				
ST-segment elevation	30 (36.1)	4 (28.6)	26 (37.7)	0.518
Ventricular tachycardia or ventricular fibrillation	12 (14.5)	5 (35.7)	7 (10.1)	0.026
Atrioventricular block	14 (16.9)	2 (14.3)	12 (17.4)	1.000
ALT(U/L) Median [Q1, Q3]	85 [44,408]	286 [48,1379]	70 [42.5,318]	0.128
Crea(umol/l) Median [Q1, Q3])	74 [57,116]	160 [83,331]	71 [48,101]	0.001
Vasoactive agent n;(%)	60 (72.3)	14 (100)	46 (66.7)	0.008
Anti-viral therapy n;(%)	81 (97.6)	12 (85.7)	69 (100)	0.027
Glucocorticoid therapy n;(%)	83 (100)	14 (100)	69 (100)	1.000
IVIG n;(%)	73 (88.0)	8 (57.1)	65 (94.2)	0.001
Mechanical ventilation n;(%)	32 (38.6)	7 (50.0)	25 (36.2)	0.335
IABP n;(%)	67 (80.7)	7 (50.0)	60 (87.0)	0.004
ECMO n;(%)	19 (22.9)	3 (21.4)	16 (23.2)	1.000
IABP + ECMO n;(%)	17 (20.5)	3 (21.4)	14 (20.3)	1.000

*CMR* cardiac magnetic resonance, *ECG* electrocardiogram, *ALT* Alanine aminotransferase, *Crea* creatinine, *IVIG* Intravenous immunoglobulin, *IABP* Intraaortic balloons pump, *ECMO* extracorporeal membrane oxygenation

### Baseline and tendency of hs-cTnI

**Fig. 2:**
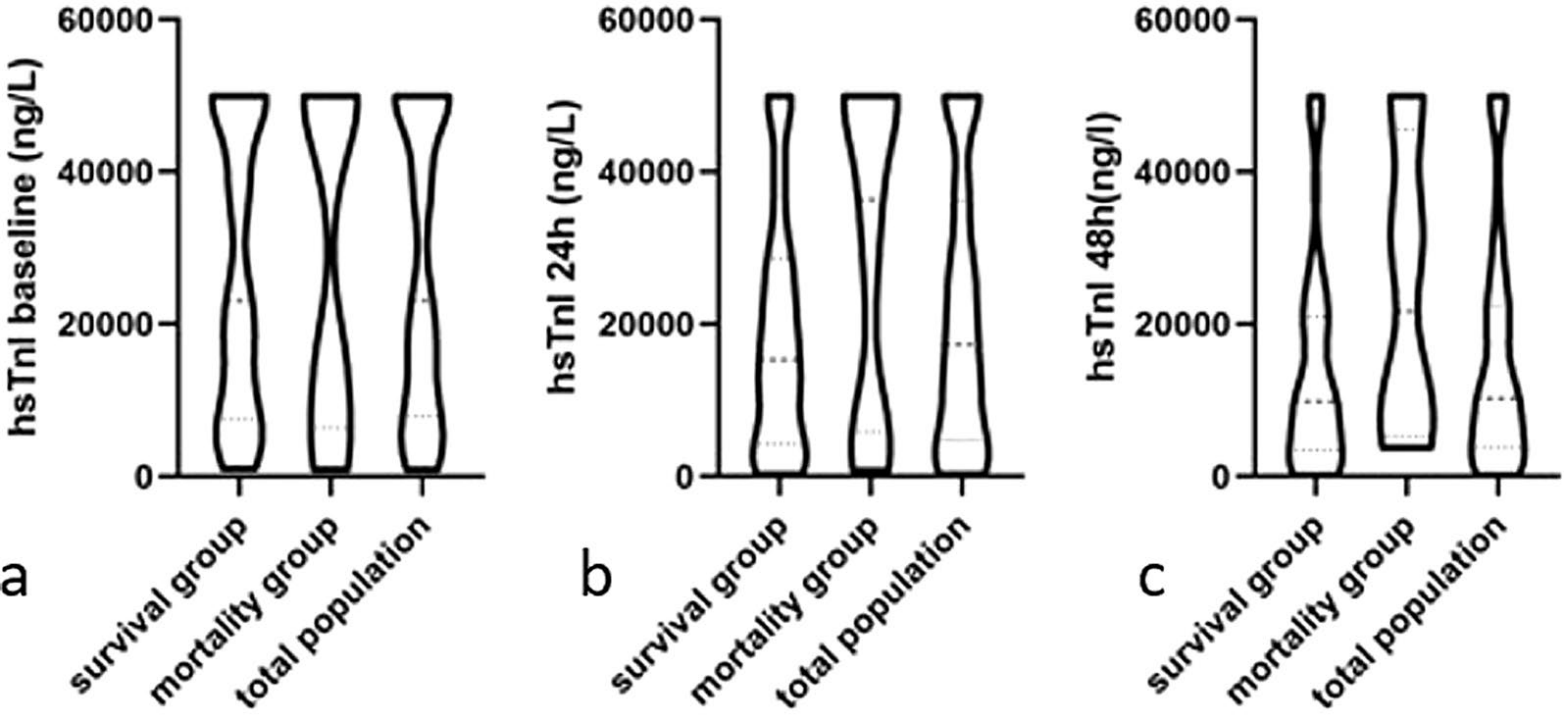
Description of hs-cTnI at baseline and within 24 h and 48 h. **a** the concentration of hs-cTnI at baseline in the three group. **b** the concentration of hs-cTnI within 24 h in the three group. **c** the concentration of hs-cTnI within 48 h in the three group

The concentration of hs-cTnI at baseline and within 24 h and 48 h were illustrated in Fig. 2., which revealed no significant difference between the mortality and survival groups at baseline concentration (survival group: 23000[7610, 50000]ng/l; mortality group: 28519[6430,50000] ng/l. *P* = 0.858). Neither of hs-cTnI within 24 h nor hs-cTnI within 48 h showed significant difference between survival group and mortality group.(hs-cTnI 24 h survival group:15335[4290,28605]ng/l versus mortality group: 36385[5928,50000]ng/l, *P* = 0.104; hs-cTnI 48 h survival group: 9904[3596,20993]ng/l versus mortality group: 21699[5380,45555] ng/l, *P* = 0.111). Levels of hs-cTnI tested within 24 h lower than levels of hs-cTnI at baseline was defined as declining a tendency; otherwise, it was defined as a non-declining tendency. There is the significant difference in the tendency of hs-cTnI change in the mortality and surviving FM patients within 24 h after admission. In the survival group, 78% of patients experienced a decrease in hs-cTnI, while 36% of the mortality group had a decreased tendency of hs-cTnI (*P* = 0.003).

### Absolute and relative hs-cTnI changes within 24 h and 48 h

**Fig. 3:**
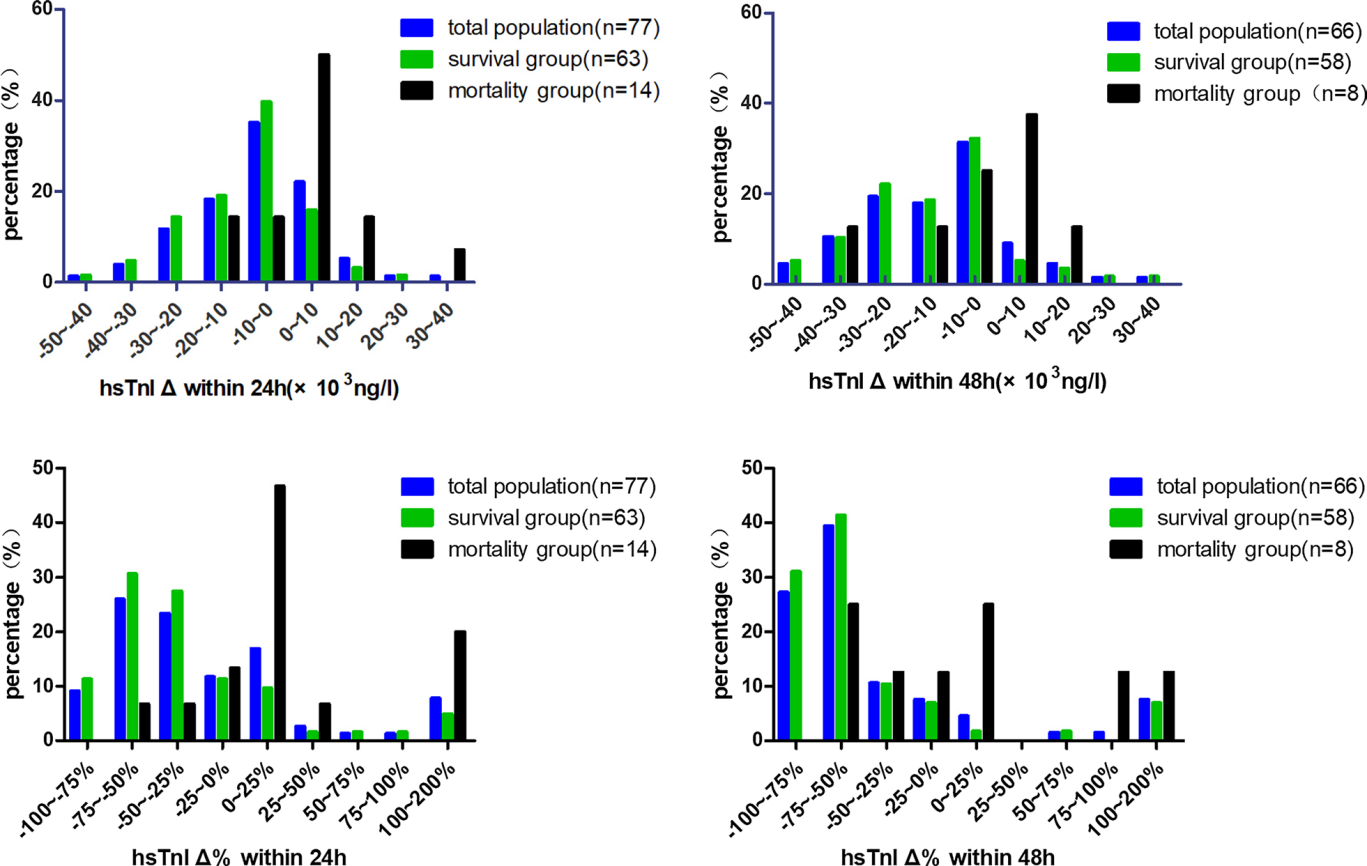
Proportion distribution of hs-cTnI Δ and hs-cTnI Δ% within 24 h and 48 h. The total population (blue), survival group (green), and mortality group (black) are displayed. hs-cTnI Δ24h: Absolute change in hs-cTnI within 24 h; hs-cTnI Δ% 24 h: relative change in hs-cTnI within 24 h; hs-cTnI Δ48h: absolute change in hs-cTnI within 48 h; hs-cTnI Δ%48 h: relative change in hs-cTnI within 48 h

The absolute and relative changes in hs-cTnI within 24 h and 48 h are illustrated in Table 2 and Fig. 3, the violin plot of hs-cTnI changes is shown in Additional file 1: Figure S1. Nearly 60% of survival group had a 0–2000 ng/l reduction in troponin from baseline within 24 h of admission. However, troponin levels of 50% of patients in the mortality group were 0–10,000 ng/ L higher than baseline 24 h after admission. And survival group patients with a relative change in hs-cTnI within 48 h between − 50 and − 100% accounted for 72.41%. The main scale of hs-cTnI distribution in the survival group and mortality group within both 24 h and 48 h was significantly different.

**Table 2:** Distribution of hs-cTnI change in survival group and mortality group within 24 h and 48 h

	Survival group proportion with 24 h	Mortality group proportion within 24 h	*P*	Survival group proportion within 48 h	Mortality group proportion within 48 h	*P*
	% (n = 63)	% (n = 14)		% (n = 58)	% (n = 8)	
Absolute change of hs-cTnI (*10^3^ ng/l)						
− 50 to − 40	1.59	0	1.000	5.08	0	1.000
− 40 to − 30	4.76	0	1.000	10.17	12.50	1.000
− 30 to − 20	14.29	0	0.198	22.03	0	0.338
− 20 to − 10	19.05	14.29	1.000	18.64	12.50	1.000
− 10 to 0	39.68	14.29	0.120	32.20	25.00	1.000
0 to 10	15.87	50.00	0.011*	5.08	37.50	0.019*
10 to 20	3.17	14.29	0.149	3.39	12.50	0.321
20 to 30	1.59	0	1.000	1.69	0	1.000
30 to 40	0	7.14	0.182	1.69	0	1.000
Relative change of hs-cTnI						
− 100 to − 75%	11.29	0	0.334	31.03	0	0.095
− 75 to − 50%	30.65	6.67	0.098	41.38	25.00	0.464
− 50 to − 25%	27.42	6.67	0.170	10.34	12.50	1.000
− 25 to 0%	11.29	13.33	1.000	6.90	12.50	0.487
0 to 25%	9.68	46.67	0.002*	1.72	25.00	0.037*
25 to 50%	1.61	6.67	0.354	0	0	–
50 to 75%	1.61	0	1.000	1.72	0	1.000
75 to 100%	1.61	0	1.000	0	12.50	0.121
100 to 200%	4.84	20.00	0.084	6.90	12.50	0.487

*Means *P* value less than 0.05

### Univariable and multivariable logistic analysis for outcome of FM

Univariable analysis was performed to select factors potentially associated with the in-hospital mortality of FM, and the results are displayed in Table 3. Six variables showed statistical significance, including the time from onset to hospital admission, occurrence of ventricular tachycardia or ventricular fibrillation, abnormal level of creatinine, declining tendency of hs-cTnI within 24 h, intravenous immunoglobulin (IVIG) treatment and IABP operation. Those variables were included in multivariable analysis. The results show that the declining tendency of hs-cTnI within 24 h, in addition to abnormal creatinine, IVIG treatment and time from onset to hospital admission were associated with the outcome of FM (for time from onset to hospital, OR = 1.3, 95% CI 1.02–1.75, *P* = 0.04; for abnormal creatinine, OR = 7.85, 95% CI 1.19–51.74, *P* = 0.032; for the declining tendency of hs-cTnI within 24 h, OR = 0.10, 95% CI 0.02–0.68, *P* = 0.018; for IVIG treatment, OR = 0.028, 95% CI 0.003–0.3, *P* = 0.003).

**Table 3:** Univariable and multivariable logistic analysis for in-hospital mortality of FM

Factor	Univariable analysis		Multivariable analysis	
	OR (95%CI)	*P*	OR (95% CI)	*P*
Female	0.73 (0.23–2.32)	0.592		
Age (year)	1.02 (0.98–1.07)	0.321		
Premonitory symptoms of viral infection	1.33 (0.37–4.80)	0.668		
Time from onset to admission	1.22 (1.03–1.45)	0.023	1.34 (1.02–1.75)	0.038
Chest pain	1.96 (0.40–9.66)	0.407		
Chest distress	0.91 (0.26–3.25)	0.890		
Disturbances of consciousness	1.02 (0.25–4.12)	0.979		
Ejection fraction at lowest %				
ST-segment elevation	0.66 (0.19–2.33)	0.520		
Ventricular Tachycardia or Ventricular fibrillation	4.92 (1.28–18.86)	0.020	6.68 (0.87–51.04)	0.067
Atrioventricular block	0.79 (0.16–4.00)	0.778		
ALT	1.33 (0.38–4.70)	0.655		
Crea	9.82 (2.68–36.02)	0.001	7.85 (1.19–51.74)	0.032
IVIG	0.08 (0.02–0.35)	0.001	0.03 (0.003–0.3)	0.003
Mechanical ventilation	1.76 (0.55–5.60)	0.338		
IABP	0.150 (0.04–0.53)	0.003	–	–
ECMO	0.90 (0.22–3.64)	0.886		
IABP + ECMO	1.07 (0.26–4.37)	0.923		
Tendency of hs-cTnI within 24 h	0.09 (0.02–0.32)	< 0.01	0.1 (0.02–0.68)	0.018

*ALT* Alanine aminotransferase, *Crea* creatinine, *IVIG* Intravenous immunoglobulin, *IABP* Intraaortic balloons pump, *ECMO* extracorporeal membrane oxygenation

### Value of absolute hs-cTnI change and relative hs-cTnI change in predicting in-hospital mortality

**Fig. 4:**
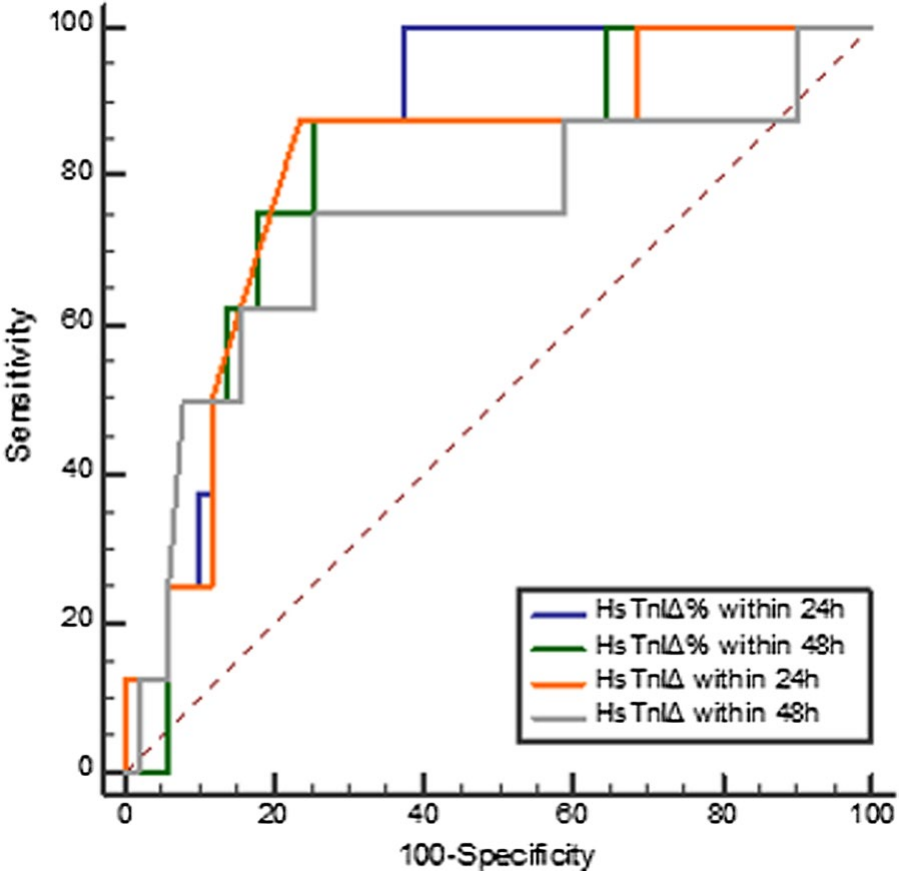
prognostic value of hs-cTnI Δ24 h, hs-cTnI Δ48 h, hs-cTnI Δ%24 h, and hs-cTnI Δ%48 h for hospital mortality of patients with fulminant myocarditis. hs-cTnIΔ24 h (orange): AUC = 0.800; hs-cTnIΔ48 h (grey): AUC = 0.711; hs-cTnIΔ%24 h (blue): AUC = 0.810; hs-cTnI Δ%48 h (green): AUC = 0.795

**Fig. 5:**
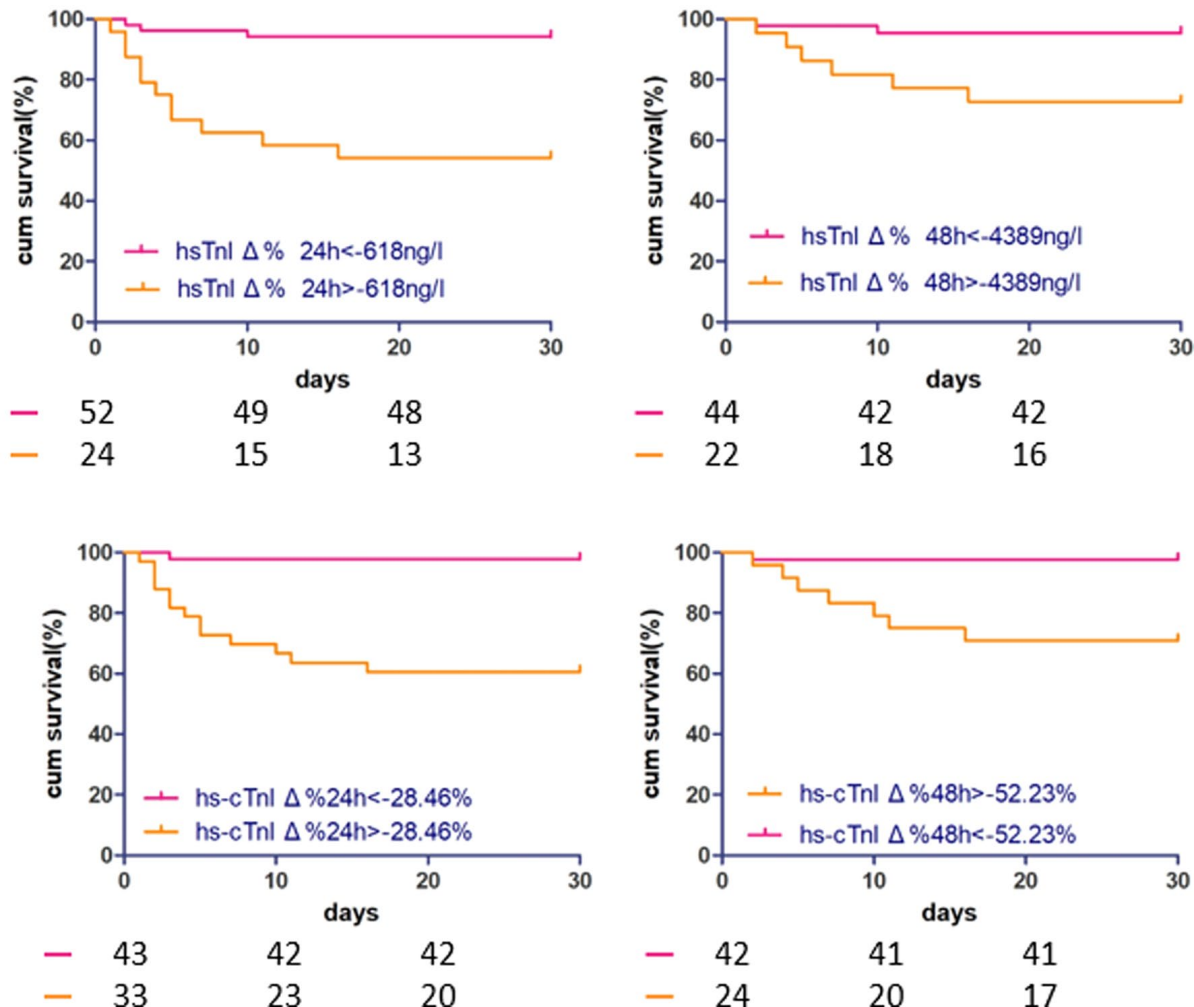
K–M analysis curve for in-hospital mortality of FM according to cut-off point. **a** K–M analysis curve of hs-cTnIΔ24 h grouped by cut-off of − 618 ng/l. **b** K–M analysis curve of hs-cTnIΔ48 h grouped by cut-off of − 4389 ng/l. **c** K–M analysis curve of hs-cTnIΔ%24 h grouped by cut-off of – 28.46%. **d** K–M analysis curve of hs-cTnIΔ%48 h grouped by cut-off of − 52.23%. Log-rank value in all models were less than 0.05

ROC curves were generated to calculate the cut-off as well as to evaluate the value of absolute and relative hs-cTnI changes in predicting the in-hospital mortality of FM. The results, including the cut-off, area under the curve (AUC), and 95% confidence interval, are displayed in Fig. 4 and Table 4, showing that the predictive value of absolute hs-cTnI changes for in-hospital mortality of FM was high both within 24 h (cut-off = − 618 ng/l; AUC, 0.80; 95% confidence interval, 0.692 to 0.883) and within 48 h (cut-off = − 4389 ng/l; AUC, 0.711; 95% confidence interval, 0.587–0.816). The accuracy of the relative hs-cTnI change for predicting FM in-hospital mortality also high both within 24 h (cut-off = − 28.46%; AUC, 0.81; 95% confidence interval, 0.704 to 0.891) and within 48 h (cut-off =  − 52.23%; AUC, 0.795; 95% confidence interval, 0.678–0.885). There was no significant difference among the relative change and absolute change at either time point (*P* > 0.05 for comparison; Fig. 4 and Table 3). According to the cut-off point of absolute and relative change of hs-cTnI within 24 h and 48 h, the whole FM patients was divided into two groups respectively. The 30 days survival of patients, grouped by the cut-off value of relative and absolute change of hs-cTnI within 24 h and 48 h, were compared. And the four kinds of K–M analysis curve was illustrated as Fig. 5. The result showed that all of the log-rank value in the four model is less than 0.05, indicating that the cut-off point of hs-cTnI change may predict the in-hospital mortality of FM. Cox regression analysis for mortality in FM patients grouped by cut-off of relative and absolute changes in hs-cTnI within 24 h and 48 h are shown in Table 5. Absolute change in hs-cTnI and relative change in hs-cTnI within both 24 h and 48 h in addition to elevated creatinine were strong predictors of in-hospital mortality, after adjustment for sex, time from onset to hospital admission, occurrence of ventricular tachycardia and ventricular fibrillation (hs-cTnI_Δ24h_ > − 618 ng/l: HR = 6.93 [1.88–25.64], *P* = 0.004; hs-cTnI_Δ48h_ > − 4389 ng/l: HR = 6.47 [1.29–32.42], *P* = 0.023; hs-cTnI_Δ%24 h_ > − 28.46%: HR = 13.83 [1.76–108.95], *P* = 0.013; hs-cTnI_Δ%48 h_ > − 52.23%: HR = 19.88 [2.40–164.79], *P* = 0.006).

**Table 4:** Diagnostic value of hs-cTnI Δ24h, hs-cTnI Δ48h, hs-cTnI Δ%24 h, hs-cTnI Δ%48 h for in-hospital mortality of patients with fulminant myocarditis

	AUC	95% CI	Cutoff
hs-cTnI _Δ24h_	0.800	0.692–0.883	− 618 ng/l
hs-cTnI _Δ48h_	0.711	0.587–0.816	− 4389 ng/l
hs-cTnI _Δ%24 h_	0.810	0.704–0.891	− 28.46%
hs-cTnI _Δ%48 h_	0.795	0.678–0.885	− 52.23%

**Table 5:** Cox proportional hazards analysis for mortality in FM

	HR	95% CI	*P* value
*Model 1*			
hs-cTnI_Δ24h_ > −0.618 ng/ml	6.93	1.88–25.64	0.004
Elevated creatinine	4.63	1.41–15.19	0.011
*Model 2*			
hs-cTnI _Δ48h_ > −4.389 ng/ml	6.47	1.29–32.42	0.023
Elevated creatinine	19.03	2.32–155.85	0.006
*Model 3*			
hs-cTnI _Δ%24 h_ > −28.46%	13.83	1.76–108.95	0.013
Elevated creatinine	4.04	1.24–13.15	0.020
*Model 4*			
hs-cTnI _Δ%48 h_ > −52.23%	19.88	2.40–164.79	0.006
Elevated creatinine	27.20	3.28–225.40	0.002

All models were adjusted for gender, time from onset to admission, occurrence of ventricular tachycardia or ventricular fibrillation

## Discussion

The main findings of this study include three aspects: (1) to the best of our knowledge, this study was the first to describe the tendency and extent of hs-cTnI change within 24 h and 48 h after admission in FM patients; (2) we determined that the tendency of hs-cTnI change within 24 h was associated with in-hospital mortality of FM; and (3) we further determined that the extent of the absolute change and relative change in hs-cTnI within 24 h and 48 h were significant factors for the prediction of the in-hospital mortality of FM.

Detection of hs-cTnI at admission is recommended in myocarditis guidelines [10, 19] because almost all patients showed elevated levels of troponin. Enrico Ammirati conducted a cohort study comprising 118 myocarditis patients with left ventricular ejection fraction (LVEF) less than 50% and 325 myocarditis patients with no such complications, and more than 99% of the patients in both groups had increased troponin levels at admission [20]. Little research has focused on the relationship between serial changes in cardiac troponin and the in-hospital mortality of myocarditis, the majority of which have explored hs-cTnI baseline prognostic value. Studies have demonstrated that the level of cardiac troponin is not associated with the outcome [21–23]. In contrast, other studies reported that elevated troponin in the early phase of myocarditis was associated with ECMO or worse prognosis [24, 25]. The prognostic value of baseline cardiac troponin in myocarditis is still debated.

Although the increased level of hs-cTnI in FM is well recognized, the tendency and exact extent of hs-cTnI change in reality remain unknown. This study described the situation of hs-cTnI change, revealing that it is normal for hs-cTnI to change in FM patients. Most surviving patients experienced a decline in hs-cTnI within 24 h, while few patients in the mortality group had decreased hs-cTnI, which was significantly different from the survival group. Within 24 h after admission, 40% of surviving patients had absolute reductions in hs-cTnI of 0–10,000 ng/l, and 58% of surviving patients showed relative reductions in hs-cTnI of 25–75%. Patients in the mortality group had absolute increases in hs-cTnI of 0–10,000 ng/l, accounting for 50% of patients. The depiction of hs-cTnI changes in FM patients is of importance for us to better understand fulminant myocarditis and provide better treatment for patients.

In addition, the declining tendency of hs-cTnI within 24 h was found to be associated with the in-hospital outcome of FM patients after adjustment for abnormal creatinine levels, IVIG treatment and time from onset to hospital stay. The declining tendency may reveal that treatment for FM in our centre was effective, including antiviral therapy, immunomodulatory therapy, circulation support, and respiratory support, resulting in the relief of inflammatory reactions that caused myocardial injury and a reduction in the incidence of ventricular tachycardia or ventricular fibrillation. In contrast, the opposite tendency may indicate that the inflammatory reaction was still severe after treatment, resulting in a high incidence of arrhythmia.

Furthermore, the prognostic value of the extent of hs-cTnI change, as absolute change and relative change, within 24 h and 48 h was explored. The results revealed that the concrete absolute and relative changes within 24 h and 48 h were associated with the in-hospital mortality of FM. Specifically, if hs-cTnI in FM patients within 24 h dropped by 618 ng/l or 28.46%, it is more likely for them to survive, which can also be predicted by the absolute change in hs-cTnI within 48 h of -4389 ng/l or relative decline of 52.23%. This result is beneficial for doctors to evaluate treatment regimens and patient prognoses. If patients exhibit a declining tendency within 24 h with the magnitude reaching our proposed cut-off value, the outcome may be good. However, if a patient's hs-cTnI change within 24 h showed an increasing tendency or the magnitude dropped, not reaching the cut-off value, it could remind doctors to reassess the treatment for better control of the inflammatory reaction.

There are still some limitations in the current study. The retrospective nature of this research may have introduced potential bias. Although we included all FM patients in our single centre during a period of 10 years, the number of cases was not sufficient for further subanalysis. The median time from onset to hospital admission was three days; thus, the peak time of hs-cTnI was undetermined for patients who experienced a declining tendency of hs-cTnI after admission. It should be noted that the criteria for patient inclusion were different, and the treatment content of fulminant myocarditis is still controversial, such as the dosage of hormones and the use of antiviral drugs. All these factors have an offset effect on the research results. Prospective research of more regular and frequent detection of hs-cTnI needs to be performed to better understand hs-cTnI changes in FM patients.

## Conclusion

Most surviving FM patients have a declining tendency of hs-cTnI under treatment within the early phase. The absolute and relative changes in hs-cTnI within 24 h and 48 h have significant predictive value for the in-hospital mortality of FM.

## Supplementary Information

**Additional file 1**. The violin plot of hs-cTnI Δ and hs-cTnI Δ% within 24 h and 48 h. **a**. the violin plot of absolute change in hs-cTnI within 24h; **b**. the violin plot of relative change in hs-cTnI within 24h; **c**. the violin plot of absolute change in hs-cTnI within 48h; **d**. the violin plot of relative change in hs-cTnI within 48h.

## Data Availability

The information and data of the study population were acquired from Hospital Information System and were recorded manually in EXCEL to form the database. The datasets analyzed during the current study are not publicly available due to the protection of the individual privacy but are available from the corresponding author on reasonable request.

## Abbreviations

hs-cTnI: High-sensitivity troponin I
FM: Fulminant myocarditis
ROC: Receiver operating characteristic
AUC: Area under curve
HF: Heart failure
EMB: Endomyocardial biopsy
CMR: Cardiac magnetic resonance
LGE: Late gadolinium enhancement
cTn: Cardiac troponin
MI: Myocardial infraction
IABP: Intraaortic balloons pump
ECMO: Extracorporeal membrane oxygenation
IVIG: Intravenous immunoglobulin
LVEF: Left ventricular ejection fraction
