# Supplementary material for: The absolute and relative changes in high-sensitivity cardiac troponin I are associated with the in-hospital mortality of patients with fulminant myocarditis
Source: BMC Cardiovasc Disord. 2021 Nov 30;21:571. doi: 10.1186/s12872-021-02386-8 (PMC8638250; doi:10.1186/s12872-021-02386-8)
Supplement: Supplementary file 1 — Additional file 1. The violin plot of hs-cTnI Δ and hs-cTnI Δ% within 24 h and 48 h. a. the violin plot of absolute change in hs-cTnI within 24h; b. the violin plot of relative change in hs-cTnI within 24h; c. the violin plot of absolute change in hs-cTnI within 48h; d. the violin plot of relative change in hs-cTnI within 48h. [file 12872_2021_2386_MOESM1_ESM.docx]

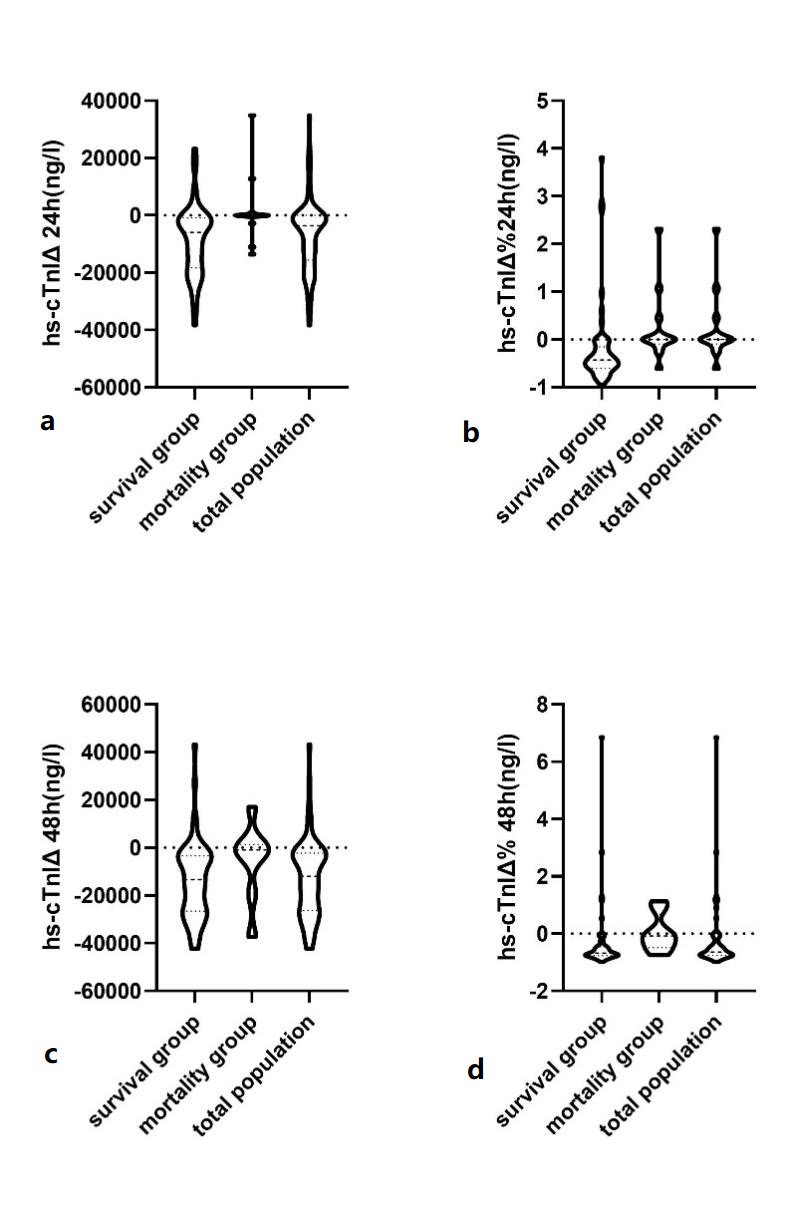


Figure title: supplemental file 1. The violin plot of hs-cTnI Δ and hs-cTnI Δ% within 24 h and 48 h

Figure legend: a. the violin plot of absolute change in hs-cTnI within 24h; b. the violin plot of relative change in hs-cTnI within 24h; c. the violin plot of absolute change in hs-cTnI within 48h; d. the violin plot of relative change in hs-cTnI within 48h.
